# Primary Care Provider Preferences Regarding Artificial Intelligence in Point-of-Care Cancer Screening

**DOI:** 10.1177/23814683251329007

**Published:** 2025-04-04

**Authors:** Vinayak S. Ahluwalia, Marilyn M. Schapira, Gary E. Weissman, Ravi B. Parikh

**Affiliations:** Perelman School of Medicine, University of Pennsylvania, Philadelphia, PA, USA; Leonard Davis Institute of Health Economics, University of Pennsylvania, Philadelphia, PA, USA; Leonard Davis Institute of Health Economics, University of Pennsylvania, Philadelphia, PA, USA; Department of Medicine, University of Pennsylvania, Philadelphia, PA, USA; Leonard Davis Institute of Health Economics, University of Pennsylvania, Philadelphia, PA, USA; Department of Medicine, University of Pennsylvania, Philadelphia, PA, USA; Palliative and Advanced Illness Research (PAIR) Center, University of Pennsylvania Perelman School of Medicine, Philadelphia, PA, USA; Emory University School of Medicine, Atlanta, GA, USA; Emory Winship Cancer Institute, Atlanta, GA, USA

**Keywords:** artificial intelligence, primary care, cancer screening

## Abstract

**Background.** It is unclear how to optimize the user interface and user experience of cancer screening artificial intelligence (AI) tools for clinical decision-making in primary care. **Methods.** We developed an electronic survey for US primary care clinicians to assess 1) general attitudes toward AI in cancer screening and 2) preferences for various aspects of AI model deployment in the context of colorectal, breast, and lung cancer screening. We descriptively analyzed the responses. **Results.** Ninety-nine surveys met criteria for analysis out of 733 potential respondents (response rate 14%). Ninety (>90%) somewhat or strongly agreed that their medical education did not provide adequate AI training. A plurality (52%, 39%, and 37% for colon, breast, and lung cancers, respectively) preferred that AI tools recommend the interval to the next screening as compared with the 5-y probability of future cancer diagnosis, a binary recommendation of “screen now,” or identification of suspicious imaging findings. In terms of workflow, respondents preferred generating a flag in the electronic health record to communicate an AI prediction versus an interactive smartphone application or the delegation of findings to another healthcare professional. No majority preference emerged for an explainability method for breast cancer screening. **Limitations.** The sample was primarily obtained from a single health care system in the Northeast. **Conclusions.** Providers indicated that AI models can be most helpful in cancer screening by providing prescriptive outputs, such as recommended intervals until next screening, and by integrating with the electronic health record. **Implications.** A preliminary framework for AI model development in cancer screening may help ensure effective integration into clinical workflow. These findings can better inform how healthcare systems govern and receive reimbursement for services that use AI.

**Highlights:**

- Clinicians do not feel their undergraduate or graduate medical education has properly prepared them to engage with AI in patient care.
- We provide a preliminary framework for deploying AI models in primary care–based cancer screening.
- This framework may have implications for health system governance and provider reimbursement in the age of AI.

Artificial intelligence (AI) and machine learning (ML) have shown promise in cancer screening. Several models have demonstrated strong predictive performance in identifying patients at high risk for colorectal,^1–4^ breast,^5–8^ and lung^9–12^ cancers. Previous attempts to understand physician perceptions of AI-based tools have revealed generally positive sentiments, with respondents optimistic that AI can improve diagnostic capabilities and efficiency in clinical workflows.^13–16^ Common concerns raised included the lack of explainability in recommendations from ML models and poor interpretability of model uncertainty.^17–21^ Amann et al.^22^ describe explainability as “a characteristic of an AI-driven system allowing a person to reconstruct why a certain AI came up with the presented predictions.”

Given the contribution of primary care providers (PCPs) toward decreased cancer morbidity and mortality,^23–25^ there is an opportunity to optimize the use of AI in cancer screening in the primary care setting. We developed a conceptual model to guide this study. Before the advent of AI, providers used a shared decision-making framework with their patients^26–29^ with multiple information sources such as 1) evidence-based population-based guidelines, such as those from the United States Preventative Services Task Force (USPSTF)^30^; 2) the patient’s individual risk factors for cancer; and 3) patient values and preferences^31–34^ (Figure 1). Using these 3 criteria, clinicians could risk stratify their patients, order additional diagnostic workup, and/or schedule their patient’s next cancer screening. AI-based clinical decision tools offer a new source of information but it is unclear how software developers and health systems should deploy these tools to meaningfully inform clinical decision making.^24^

**Figure 1:**
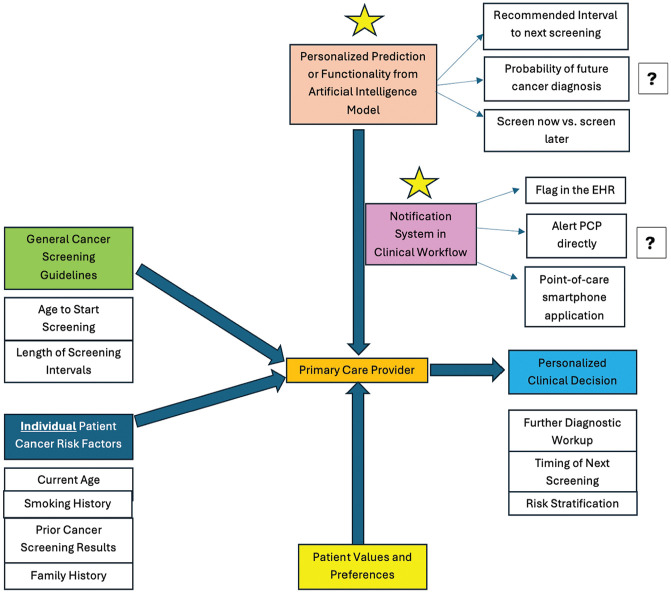
Conceptual model depicting the interplay between individual patient risk factors, general population-based guidelines, previous cancer screening, patient preferences, and a hypothetical AI model when making a personalized cancer screening recommendation in the primary care setting. The AI model outputs a personalized prediction regarding cancer screening, the result of which could be communicated to the primary care provider (PCP) in a variety of ways (“notification system in clinical workflow”). This study aims to determine which specific prediction or functionality will best inform personalized cancer screening and how to implement AI technology in the clinical workflow. These aims are labeled with a gold star. Arrows represent the conduit by which information is communicated to the PCP so a clinical decision can be made.

Knowledge gaps that must be addressed for software developers include whether to create AI models that generate neutral statements, such as an individual patient’s absolute 10-y probability of colorectal cancer, versus models that generate prescriptive outputs, such as the recommended time until a specific patient’s next colonoscopy. Given the “black box” nature of many AI tools, it is also unclear how models should justify their outputs, which has implications for clinical decision-making and clinician trust.^35^ Lastly, due to clinicians’ busy patient schedules and documentation responsibilities, these tools cannot reach their potential if health systems do not appropriately deploy them in the clinical workflow.^36,37^ It remains an open question how to ensure these tools are an asset in clinical workflow, rather than a hindrance or distraction.

This survey study aims to describe the general attitudes and preferences of PCPs regarding personalized AI-based clinical decision tools for which the USPSTF has reported population-based cancer screening guidelines: colorectal, breast, and lung cancers. Answering these questions will inform a preliminary framework for the user interface and user experience (UI/UX) of such tools in the primary care setting. A standardized framework for AI tools not only increases the likelihood of better patient care but also carries consequences for healthcare policy and practice.

## Methods

### Eligibility Criteria and Study Design

We surveyed attending physicians, family medicine residents, and advanced practice providers (APPs) using an electronic survey instrument developed on the Qualtrics (Provo, UT, USA) platform. Participants were eligible to complete the form if they were attending physicians or APPs practicing in primary care or if they were family medicine residents. We distributed the survey to providers fitting this criterion at the Hospital of the University of Pennsylvania (HUP), an urban academic medical center, and Lancaster General Hospital (LGH), a regional hospital affiliated with the University of Pennsylvania. We also employed convenience sampling to distribute the survey to other PCPs within our professional network in the United States. All attending physicians or APPs had trained in internal medicine or family medicine. This study was exempt from approval of the institutional review board at the University of Pennsylvania.

### Instrument Development

The survey was separated into 3 sections: 1) demographics and background of respondents; 2) general attitudes and preferences towards AI, including the expected impacts of AI on health care utilization related to cancer screening; 3) preferences for prediction outputs for hypothetical AI models, implementation of such models in clinical workflow, and methods of AI model explainability assessed with clinical vignettes (Appendix). Questions and clinical vignettes were informed by our conceptual model of clinical decision-making (Figure 1), expert input, and prior literature on attitudes toward AI. The survey was piloted and iteratively revised based on primary care and specialty clinician feedback before dissemination.

### Terminology

Given the potential of care managers and nurse navigators to provide patient-centered care as a part of a large multidisciplinary health care team^38–42^ as well as the study team’s personal experience working with such individuals in oncology care, we wished to know if care managers could help deploy AI tools. We defined the term “licensed care manager” as a “non-physician licensed healthcare provider who can directly coordinate additional lab work or imaging studies with a patient.” We intentionally did not define AI or ML in the survey introduction because we wished to understand respondents’ preconceived notions about AI and ML. When referencing “AI” in the survey, “AI” encompassed both AI and ML tools.

### General Demographics and Respondent Background

Section 1 of the survey included the participant’s date of birth, health care role, medical specialty, presence of fellowship training (if applicable), years in independent practice, whether the participant had spent at least 50% of their clinical time in a primary care context in the last year, and whether the participant had previously used AI tools in a clinical context.

### Attitudes toward AI and ML

Section 2 of the survey contained 15 Likert scale–based questions regarding general attitudes toward AI and/or ML (henceforth referred to as “AI”) using a 5-point response scale of *strongly disagree*, *somewhat disagree*, *neither agree nor disagree*, *somewhat agree*, and *strongly agree*. The domains covered in this section were prior understanding of AI, patient safety and privacy, previous experience with AI in medical training, impact on the primary care workforce, and the predicted impact of AI on health care utilization related to cancer screening (in terms of costs and unnecessary diagnostic procedures). One question asked respondents to select all entities that should bear responsibility for regulating AI, including the federal government (such as the Food and Drug Administration), state governments, physician organizations, software developers, and individual providers. We surveyed respondents about their medical training and AI regulation since these perceptions may influence preferences toward AI in cancer screening.

### Clinical Vignette Questions

Section 3 was composed of 11 clinical vignettes that asked participants to assume the role of a PCP and report various preferences regarding AI in the context of cancer screening decisions (see Appendix).

Vignettes 1 to 3 asked the respondent to select the preferred prediction metric or functionality for a hypothetical AI model to communicate a cancer screening recommendation. Options provided were a 5-y probability of a cancer diagnosis, recommended time until the next screening test should be ordered, whether to screen now or defer to the next USPSTF-recommended interval, or not using any of these tools. Participants were given the same scenario in each vignette but the type of cancer screening test in question (colorectal, breast, or lung cancer) varied. An example is seen below:

You are a primary care provider who has just learned that your health system is deciding between purchasing a license to one of four FDA-approved AI-based tools for colorectal cancer screening management. This tool only applies to patients who have received at least one colonoscopy in the past. Assume that all tools have comparable accuracy.

Vignettes 4 to 6 asked the respondents to choose how best to implement an AI tool in the clinical workflow for cancer screening. We sought to examine the clinical workflow because previous work has suggested that automated alert systems can improve patient follow-up and closed-loop communication in radiology contexts,^36,37,43–45^ although it is unclear if the electronic health record (EHR) is the preferred avenue for communicating AI recommendations in the primary care setting. The following scenarios were identical for each vignette but varied in cancer type (colorectal, breast, or lung). Response options included 1) generating a flag in the EHR for eligible patients, 2) having an accessible risk calculator online or in a smartphone application, 3) providing predictions to a practice’s licensed care manager once per year, or 4) none of the above. An example scenario is seen below:

You are a primary care provider who has just learned that your health system is purchasing a license for a new tool that estimates the five-year risk of colorectal cancer ([0-100%] with 95% CI) for each patient of screening age, regardless of previous screenings. The health system is deciding how best to implement the tool in clinical workflow. Please select how you would prefer to implement the tool’s predictions.

Vignettes 7 and 8 asked respondents which members of the healthcare team should be alerted if a hypothetical AI model detects suspicious findings on routine lung cancer or breast cancer screening imaging. Options included 1) alerting the attending radiologist, 2) alerting the patient’s PCP, 3) alerting a licensed care manager, or 4) none of the above. Participants could select multiple options if desired. The vignettes varied only in the cancer type. The order in which cancer types were presented was the same for all respondents (lung then breast). An example scenario is seen below:

Your hospital system has recently purchased a license to an AI-based tool that segments (outlines) regions that are suspicious for breast masses. The tool offers three options for how suspicious masses can be communicated to the healthcare team. Assume the patient has never been diagnosed with a cancer. Please select which members of the care team should be alerted to suspicious masses (select all that apply).

Vignettes 9 and 10 asked respondents when, in the clinical workflow, an attending radiologist should be able to view an AI-generated radiology report for screening mammograms or low-dose chest computed tomography (CT). Options included 1) the radiologist viewing the AI-generated report after viewing the imaging herself but before signing her final report, 2) the radiologist signing her final report before viewing the AI-generated report, 3) the radiologist viewing an AI-generated report before viewing the imaging, or 4) none of the above. The vignettes varied only in the cancer type; the order in which cancer types were presented was the same for all respondents (lung then breast). An example scenario is seen below:

Your hospital system has recently purchased a license to an AI-based tool that segments (outlines) regions of mammograms that are suspicious for pathologic breast masses. The tool offers three options for how suspicious breast masses can be communicated to the healthcare team. Assume the patient has never been diagnosed with a cancer. Please select which members of the care team should be alerted to suspicious breast masses (select all that apply).

Vignette 11 dealt with different methods for explainability pertaining to a breast cancer risk prediction tool that provides a binary recommendation (follow-up needed or no follow-up needed). Options included 1) model reporting the percentage probability of developing breast cancer within the next 5 years with delineation of the most relevant risk factors, 2) model estimating the volumetric breast density from the mammogram and assigning a Breast Imaging-Reporting and Data System (BI-RADS) density category^46^, 3) a model segmenting suspicious masses on previous mammograms, 4) no preference for any of these explainability methods, or 5) no preference for any of these methods. See the appendix for the graphics accompanying each response option; graphics for options 2 and 3 for this vignette were reproduced from He et al.^47^ and Hassan et al.,^48^ respectively. The following background was given:

The “explainability” of AI models is an important factor in determining provider trust of these models’ recommendations. You are a primary care provider who is piloting a new tool to aid in the management of breast cancer screening for patients who recently received a standard mammogram. The tool provides a binary recommendation (follow-up needed or no follow-up needed). The developers of this tool want to know the best way in which the tool can explain its decision-making. They provide you three options for how to accomplish this. Visual accompaniments are below.

Cancer types were presented in the same order (colorectal, breast, lung) across all vignettes, unless otherwise specified.

### Statistical Analysis

Responses were included in the analysis if at least 50% of the questions were answered. Age was calculated using the provided date of birth. Time in independent practice was categorized as ≤5 y, 6 to 10 y, 11 to 20 y, 21 to 30 y, or >30 y. Healthcare role was categorized as attending physician, advanced practice provider, or resident. We conducted a descriptive analysis of attitude items and clinical vignettes. Attitude questions were assessed on a 5-point Likert-type scale (*strongly disagree* = 1, *somewhat disagree* = 2, *neither agree nor disagree* = 3, *somewhat agree* = 4, *strongly agree* = 5). For each attitudes-related question from section 2, we reported the number of respondents who selected each Likert scale–based option and then calculated an average Likert scale value with a 95% confidence interval. For questions relating to AI regulation (section 2) and workflow (section 3), respondents could select more than 1 option, and we report the total affirmative responses for each option.

## Results

### Response Rate and Sample

We distributed the survey to 733 providers and received 99 responses from 9 different clinics that met criteria for analysis, yielding an overall response rate of 14%. The demographics of the respondents can be seen in Table 1. The median age of respondents who provided their age was 44 y (interquartile range 36–55 y). Seventy-two (73%) were attending physicians, 23 (23%) were APPs, and 2 (2%) were family medicine residents. Seventy-one (72%) were affiliated with the HUP and 18 (18%) with LGH. Nine (9%) were not affiliated with HUP or LGH, including 2 (2%) in California and 7 (7%) in Illinois. Seventy-four (75%) had been practicing independently in the primary care setting for more than 5 y. Fourteen (14%) reported using an AI-based tool in clinical practice in the past.

**Table 1:** Demographics of the Respondents

Characteristic	*N* = 99
Median age (interquartile range), y	44 (36–55)
Health care role	
Attending physician	72 (73%)
Advanced practice provider	23 (23%)
Family medicine resident	2 (2%)
No response	2 (2%)
Institutional affiliation	
Hospital of the University of Pennsylvania	71 (72%)
Lancaster General Hospital	18 (18%)
Other US-based community medical center	9 (9%)
Unsure	1 (1%)
Time spent in practice independently, y	
≤5	21 (21%)
6–10	20 (20%)
11–20	17 (17%)
21–30	24 (24%)
>30	13 (13%)
No response	4 (4%)
Previous experience with AI in the clinical setting	
Yes	14 (14%)
No	56 (57%)
Unsure/no response	29 (29%)
Completed fellowship training	
Yes	11 (11%)
No	61(62%)
No response	27 (27%)
Spent at least 50% of clinical time in primary care during the last year	
Yes	93 (94%)
No	4 (4%)
Unsure	1 (1%)
No response	1 (1%)

### Results of the Survey of Attitudes

**Figure 2:**
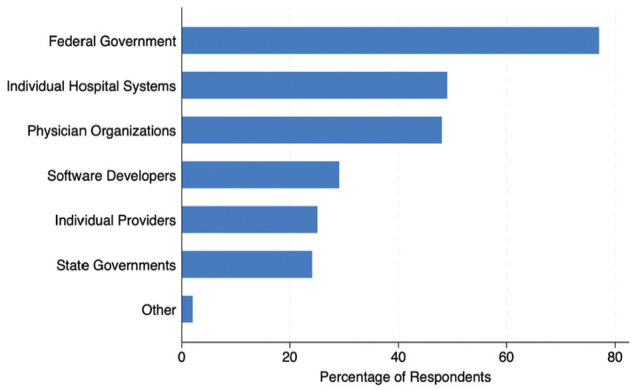
Participant opinion regarding who bears responsibility for regulating artificial intelligence (AI) in the health care field. Participants could select multiple options if desired.

Of the respondents, 73 (74%) were somewhat or strongly confident that they could explain the term *artificial intelligence* to a layperson. However, 90 (91%) and 89 (90%) of respondents felt they did not receive adequate training regarding AI in their undergraduate and graduate medical training, respectively. According to the respondents (Figure 2), entities that should have responsibility for regulating healthcare AI in the United States were the federal government (76%), individual hospital systems (49%), and physician organizations (48%). Only 25% felt that individual providers should be responsible for the AI models they used. A minority of respondents expressed concern that AI would endanger patient safety (25%) or affect the privacy of protected health information (35%).

Most respondents somewhat agreed or strongly agreed that AI tools would improve personalized screening decisions for patients at risk for colorectal cancer (65%), breast cancer (67%), and lung cancer (70%). A plurality of respondents expected AI tools to somewhat or significantly decrease the number of unnecessary colonoscopies (39%), breast biopsies (39%), and lung nodule biopsies (39%). Only 14% reported concern that the advent of AI in healthcare would negatively affect job prospects in primary care in the United States (Table 2).

**Table 2:** Experiences of Respondents Regarding AI in Their Medical Training as Well as Perceptions of Its Benefits and Risks in Health Care^a^

Statement	*Strongly Disagree*	*Somewhat Disagree*	*Neither Agree nor Disagree*	*Somewhat Agree*	*Strongly Agree*	*No Response*	*Mean Response (95% CI)*
“I could explain to a friend or family member what ‘artificial intelligence’ means.”	2 (2%)	8 (8%)	14 (14%)	50 (51%)	23 (23%)	2 (2%)	3.87 (3.67–4.06)
“My undergraduate medical training provided adequate training on AI.”	78 (79%)	12 (12%)	6 (6%)	0 (0%)	1 (1%)	2 (2%)	1.29 (1.15–1.43)
“My graduate medical training provided adequate training on AI.”	76 (77%)	13 (13%)	5 (5%)	2 (2%)	0 (0%)	3 (3%)	1.30 (1.17–1.44)
“I am concerned that AI will endanger patient safety.”	8 (8%)	28 (28%)	36 (36%)	20 (20%)	5 (5%)	2 (2%)	2.86 (2.65–3.06)
“I am concerned that AI tools will negatively impact the confidentiality of protected health information (PHI).”	7 (7%)	28 (28%)	27 (27%)	28 (28%)	7 (7%)	2 (2%)	3.00 (2.78–3.22)
“AI tools will improve personalized screening decisions for patients at risk for colorectal cancer.”	0 (0%)	5 (5%)	27 (27%)	46 (46%)	19 (19%)	2 (2%)	3.81 (3.65–3.98)
“AI tools will improve personalized screening decisions for patients at risk for breast cancer.”	0 (0%)	3 (3%)	27 (27%)	48 (48%)	19 (19%)	2 (2%)	3.86 (3.70–4.01)
“AI tools will improve personalized screening decisions for patients at risk for lung cancer.”	0 (0%)	3 (3%)	25 (25%)	50 (51%)	19 (19%)	2 (2%)	3.88 (3.72–4.03)
“I am concerned that AI tools will decrease the number of primary care attending physician, NP, or PA job positions in the United States.”	37 (37%)	30 (30%)	16 (16%)	12 (12%)	2 (2%)	2 (2%)	2.09 (1.87–2.32)

AI, artificial intelligence; NP, nurse practitioner; PA, physician assistant.

a Mean response = average of numerically coded Likert values excluding “no response.”

### Clinical Vignettes

With regard to the preferred recommendation format for AI algorithms in cancer screening, a plurality of respondents opted for a recommended time interval until next screening for all 3 cancers (Tables 3 and 4). Fifty-two percent chose this option for the next colonoscopy, compared with 39% for the next mammogram and 37% for low-dose chest CT.

**Table 3:** Perceptions Regarding the Future Impact of AI on the Costs of Cancer Screening and the Number of Unnecessary Diagnostic Procedures for Colorectal, Breast, and Lung Cancers.^a^

Statement	*Significantly Decrease*	*Somewhat Decrease*	*Have No Significant Effect On*	*Somewhat Increase*	*Significantly Increase*	*No Response*
Overall costs of screening						
Colorectal cancer	6 (6%)	33 (33%)	36 (36%)	22 (22%)	1 (1%)	1 (1%)
Breast cancer	6 (6%)	33 (33%)	30 (30%)	27 (27%)	1 (1%)	2 (2%)
Lung cancer	7 (7%)	32 (32%)	24 (24%)	32 (32%)	2 (2%)	2 (2%)
Number of unnecessary diagnostic procedures						
Colonoscopies	3 (3%)	49 (49%)	30 (30%)	13 (13%)	1 (1%)	3 (3%
Breast biopsies	7 (7%)	44 (44%)	25 (25%)	19 (19%)	1 (1%)	3 (3%)
Lung nodule biopsies	8 (8%)	47 (47%)	24 (24%)	16 (16%	1 (1%)	3 (3%)

a For each cancer, participants were asked to complete the following sentence: “AI- and ML-tools will ____ the overall costs of screening in the United States” and “AI- and ML-tools will ____ the number of unnecessary procedures in the United States.”

**Table 4:** Preferred Recommendation Format and Workflow Implementations for Hypothetical AI Algorithms Based on the Cancer.^a^

Survey Question of Interest	Colorectal Cancer	Breast Cancer	Lung Cancer
Format of recommendation			
5-y probability of cancer diagnosis (0%–100% with 95% CI)	16 (16%)	16 (16%)	13 (13%)
Recommended time until next cancer screening (years, with 95% CI)	51 (52%)	39 (39%)	37 (37%)
Binary recommendation of 1) screen now or 2) defer until the next USPTSF-recommended screening interval	14 (14%)	15 (15%)	19 (19%)
Segment suspicious findings on imaging	N/A	10 (10%)	13 (13%)
Would not use any of these tools	8 (8%)	9 (9%)	7 (7%)
No response	10 (10%)	10 10%)	10 (10%)
Workflow implementation			
Automatically generate EHR chart flag for eligible patients	56 (57%)	55 (56%)	56 (57%)
Have an easily accessible risk calculator hosted online or through a smartphone application	13 (13%)	12 (12%)	11 (11%)
Provide predictions to the practice’s licensed care manager once per year	8 (8%)	10 (10%)	11 (11%)
None of the above	6 (6%)	6 (6%)	5 (5%)
No response	16 (16%)	16 (16%)	16 (16%)
Communication of suspicious imaging findings to the healthcare team			
Alert attending radiologist	N/A	70 (71%)	68 (69%)
Alert PCP	N/A	53 (54%)	53 (54%)
Alert licensed care manager	N/A	27 (27%)	25 (25%)
None of the above	N/A	1 (1%)	1 (1%)
No response	N/A	15 (15%)	15 (15%)
Radiologist workflow implementation			
Attending radiologist may view AI-generated report for a given study before signing her final report. Both reports are available in the EMR.	N/A	44 (44%)	44 (44%)
Attending radiologist signs her final radiology report before the AI-generated report can be viewed. Both reports are available in the EMR.	N/A	10 (10%)	10 (10%)
Attending radiologist views the AI-generated report for a given study before viewing the study images herself. The attending then views images and signs the final report. Both reports are available in the EMR.	N/A	21 (21%)	21 (21%)
None of the above	N/A	7 (7%)	8 (8%)
No response	N/A	17 (17%)	16 (16%)

AI, artificial intelligence; CI, confidence interval; EMR, electronic medical record; N/A, not applicable; PCP, primary care provider; USPSTF, United States Preventative Services Task Force.

a Each vignette was single choice and single attribute. The preferred method of communication of imaging-related AI findings to the health care team and implementation in radiologist workflow. Low-dose chest computed tomography and mammogram for lung and breast cancers, respectively.

Concerning workflow implementation, most (56% or 57%) of the respondents preferred that a flag be generated in the electronic health record describing AI-generated cancer risk, greater than those who preferred involving a practice’s licensed care manager (8%–11%) or using a smartphone application (11%–13%). Respondents indicated a preference for hypothetical AI systems that detect suspicious nodules on mammograms or alert the attending radiologist (71%) and the patient’s PCP (54%) over a licensed care manager (27%). Similarly, for nodule detection on low-dose chest CT, 69% and 54% preferred that the AI alert the radiologist and PCP, respectively, compared with 25% for a licensed care manager.

For breast and lung cancer screening, a plurality (44%) preferred that AI interpretations of breast and lung imaging be made available after the radiologist makes her preliminary report but before providing a final attestation. This finding is in contrast to the radiologist making a final interpretation without using an AI at all (selected by 10%) or the radiologist accessing the AI interpretation before viewing the imaging herself (selected by 21%).

Finally, in terms of explainability for a breast cancer risk prediction algorithm (Table 5), while a plurality (33%) opted for a delineation of pertinent risk factors influencing the model’s prediction, 18% preferred more models that highlight suspicious findings on breast imaging, 8% preferred breast density estimations, and 23% had no preference regarding explainability.

**Table 5:** Preferences for Explainability for a Hypothetical AI Algorithm That Informs Breast Cancer Screening Risk.^a^

Explainability Method	
Model reports percentage probability of developing breast cancer within the next 5 y with delineation of the most relevant risk factors that influenced prediction	33 (33%)
Model estimates volumetric breast density from a mammogram and assigns BI-RADS density category	8 (8%)
Model segments suspicious masses on previous mammograms	18 (18%)
I have no preference for any of these explainability methods	23 (23%)
No response	17 (17%)

BI-RADS, Breast Imaging-Reporting and Data System.

a This vignette was single choice and single attribute.

## Discussion

This study fills an important knowledge gap by elucidating the user interface and user experience features that will best translate the power of AI models to cancer screening. Previous work has leveraged qualitative and quantitative methods to understand patient and provider preferences regarding AI in health care. Hendrix et al.^49^ used discrete choice experiments and qualitative interviews to understand what features would increase clinician trust in an AI method for breast tumor detection on screening mammograms. The authors found that for clinicians to trust the predictions from such an algorithm, sensitivity should be prioritized over specificity, radiologist confirmation of the AI prediction should be present, and the model should be trained on a diverse patient population.^49^ While this information is helpful, questions remain regarding the deployment of AI in the clinical context.

In our study, most clinicians reported optimism regarding the advent of AI in cancer screening, and few felt concerned about the impact of AI on their future job prospects, which aligns with previous work.^50^ Yet, clinicians do not feel that their medical training has adequately prepared them to engage with AI, indicating a need for medical education to adapt. Given that no current guidelines or regulations exist regarding how medical education should teach trainees to interact with AI tools and a literature describing a desire for a more rigorous AI curriculum,^50–54^ leaders in medical education should consider incorporating formal AI instruction into their programs.

Our findings support a preliminary framework to guide model deployment for software developers and health systems. Many previously published and/or widely publicized AI models rely on either highlighting suspicious findings in imaging,^55–57^ a process that is interpretable by humans, or risk stratification (i.e., low risk versus high risk for future cancer status).^58–60^ However, in the context of primary care–based cancer screening, respondents indicated a preference for prescriptive recommendations, such as an interval until the next screening, in contrast to more objective but less actionable assessments of risk. Developers may need to alter AI models so that they provide more prescriptive outputs, since those are more clinically actionable.

We now turn our focus to workflow implementation. Our findings suggest that health systems should focus on integrating AI models into the EHR and ensuring that clinician assessments are not initially biased by an AI-generated recommendation. These results are in concert with previous work concerning a hypothetical melanoma detection tool^21^ and AI-enabled medical devices in diabetes care,^61^ underscoring a desire for mitigating provider bias, robust user support services, and a limiting of device alerts to those that are pertinent and actionable. In addition, delegating the responsibility for following up on an AI-generated prediction from a PCP to another healthcare team member was not popular among providers. Ensuring that AI is conducive to the clinician’s busy schedule is paramount to maximizing its potential.

Interestingly, there was no clear consensus on a preferred method for explainability in a hypothetical AI model for breast cancer screening, with one-quarter explicitly having no preference for any of the methods we offered. While this contrasts with a substantial literature describing explainability’s importance,^17,19,22^ respondents may not have had sufficient AI literacy to understand the implications of the options. Some scholars have argued that the black box nature of AI should not necessitate its removal from healthcare spaces because some widely accepted clinical decisions rely on “opaque” reasoning.^62–64^ Future work should further investigate how best to communicate explainability and the need, if any, of explainability in the cancer screening context.

Our results apply to other emerging areas. While three-quarters of respondents in our study believe the onus of regulation lies with the federal government, other bodies are seen as responsible, including individual hospital systems, physician organizations, and software developers. This finding aligns with a literature indicating a lack of clarity regarding who is morally and/or legally accountable for the outcomes associated with this technology.^50,65–67^ One way in which individual healthcare systems can exercise oversight over this technology is through governance committees, an increasingly common review board at many institutions that ensures that AI tools promote usability, equity, and explainability in patient care.^68–70^ A UI/UX framework for such tools can guide the vetting and in-house regulation of AI models from potential vendors.

As payers begin to reimburse providers for care involving AI decision support, a new framework for future AI tools may affect whether payers choose to reimburse care that leverages AI, as well as whether AI is needed to meet certain quality metrics in value-based care models.^71,72^ Further, standardizing outputs for AI models may facilitate future cost-effectiveness studies.^50,73,74^

From a practice standpoint, a clear framework for AI models and improved AI training in medical education should allow the PCP to better engage the patient in shared decision-making regarding their cancer screening. If the clinician can better communicate her reasoning to the patient, this will lead to decisions that better align with a patient’s values.^27^ Previous work demonstrates reporting superior collaborative decision-making for patients with osteoarthritis who received an AI-enabled decision aid and patient education, as compared with those who received only patient education.^75^

This study has some limitations. First, our aims are primarily descriptive, and the study was not designed to infer relationships between AI preferences and respondent attributes including age, healthcare role, or time in practice or across cancer types. However, the directionality of the responses was consistent between each of the cancer types. Second, most respondents were affiliated with clinics in an academic medical center in the Northeast, which may not capture the preferences of providers in more community and/or rural clinical settings. Clinicians at an academic medical center may have more exposure to AI tools than those in a community setting,^69^ which may lead to them having a more favorable view of AI. Clinicians in academic settings who have a range of roles including clinical work, education, and research may have a lower concern of AI tools encroaching on their job prospects compared with those who are primarily focused on clinical work. Third, our examination of preferences regarding AI in radiology workflow relied on answers from PCPs rather than radiologists who play a key role in the breast and lung cancer screening process. Lastly, we restricted this study to the management of colorectal, breast, and lung cancers. Screening and detection of other conditions in the primary care setting could also benefit from a more standardized framework for AI models. Finally, we do not have access to the demographics of those who did not respond, so we cannot speculate about possible self-selection bias. Future work is needed to extend this study to a broader range of clinicians and clinical scenarios as the vignette options were not mutually exclusive nor exhaustive of all knowledge gaps present in the AI space.

In conclusion, through a survey of primary care clinicians, we identified key attitudes and preferences regarding AI in clinical decision-making for cancer screening. These findings constitute a preliminary framework for optimizing the deployment of AI tools in the primary care setting as well as in other contexts.

## Supplemental Material

sj-docx-1-mpp-10.1177_23814683251329007 – Supplemental material for Primary Care Provider Preferences Regarding Artificial Intelligence in Point-of-Care Cancer Screening Supplemental material, sj-docx-1-mpp-10.1177_23814683251329007 for Primary Care Provider Preferences Regarding Artificial Intelligence in Point-of-Care Cancer Screening by Vinayak S. Ahluwalia, Marilyn M. Schapira, Gary E. Weissman and Ravi B. Parikh in MDM Policy & Practice
