# Supplementary material for: Primary Care Provider Preferences Regarding Artificial Intelligence in Point-of-Care Cancer Screening
Source: MDM Policy Pract. 2025 Apr 4;10(1):23814683251329007. doi: 10.1177/23814683251329007 (PMC11970086; doi:10.1177/23814683251329007)

**Appendix: Survey Questions**

*We are a research team at the University of Pennsylvania’s medical school who are conducting a survey regarding clinician attitudes and preferences regarding artificial intelligence (AI) and machine learning (ML) in the screening of cancer. You have received this email because you are an attending physician, fellow, family medicine resident, or advanced practice provider who currently sees patients in a primary care setting. Our study consists of a brief (<15 minutes) survey via Qualtrics divided into three sections. The first section consists of basic demographic information and the second section contains multiple-choice questions regarding your general thoughts towards AI in cancer screening. In the last section, you will choose between hypothetical AI tools that are implemented in various cancer screening contexts.

The only identifying information recorded will be your email address and date of birth. Your participation is completely voluntary and you can stop filling out the survey at any point. We do not offer compensation for participation. This study is IRB-exempt at the University of Pennsylvania and Primary Care Research Integration Committee approved. If you have any questions before, during, or after survey completion, please do not hesitate to reach out to our team’s research coordinator at vinayak.ahluwalia@pennmedicine.upenn.edu or the principal investigator at ravi.parikh@pennmedicine.upenn.edu.

For this survey, we assume the following definition of “primary care” from healthcare.gov: “Health services that cover a range of prevention, wellness, and treatment for common illnesses. Primary care providers include doctors, nurses, nurse practitioners, and physician assistants. They often maintain long-term relationships with you and advise and treat you on a range of health-related issues. They may also coordinate your care with specialists.”

Note: we abbreviate primary care provider as "PCP".

We thank you in advance for your consideration! Your participation will help ensure that AI models are clinically meaningful and can positively impact patient care.*

**Section 1: Demographics**

1. What is your email address? _______________
2. What is your date of birth (MM/DD/YYYY)? _______________
3. What department do you belong to?
   1. CPUP IM (Internal Medicine)
   2. CPUP FM (Family Medicine)
   3. Penn Primary Care
   4. Lancaster General Hospital
   5. Unsure
   6. Not Penn-affiliated
4. I currently treat patients in a clinical setting and have spent most (>50%) of my clinical time in a "primary care" role over the past year.
   1. Agree
   2. Disagree
   3. Unsure
5. Please specify with which healthcare role you best identify:
   1. Attending physician
   2. Fellow (post-residency)
   3. Family medicine resident
   4. Nurse practitioner
   5. Physician assistant
   6. Other
   7. Unsure
   8. Prefer not to say
6. In which specialties have you completed a residency (select all that apply)?
   1. Internal Medicine
   2. Family Medicine
   3. Pediatrics
   4. OB/GYN
   5. Emergency Medicine
   6. General Surgery
   7. Radiology
   8. Other, please specify: ______
   9. None of the Above
7. Have you completed any fellowship training?
   1. Yes, I have completed fellowship training.
   2. No, I have not completed fellowship training and am not currently pursuing it.
   3. No, but I am currently completing fellowship training.
   4. Unsure
8. If you answered “Yes” to question 7, for how many years have been practicing primary care independently?
   1. I am still in training
   2. <=2 years
   3. 3-5 years
   4. 6-10 years
   5. 11-15 years
   6. 16-20 years
   7. 21-30 years
   8. >30 years

**Section 2: Attitudes**

The Food and Drug Administration (FDA) has approved 692 different AI-based tools for clinical implementation as of October 19, 2023. Have you used any of these tools to aid in your clinical decision-making before?

- 1. Yes
  2. No
  3. Unsure

1. I could explain to a friend or family member what “artificial intelligence” means.
   1. Strongly Agree
   2. Somewhat Agree
   3. Neutral
   4. Somewhat Disagree
   5. Strongly Disagree
2. My undergraduate medical training (medical school, NP school, PA school) provided adequate training regarding the use of AI in healthcare, including the benefits and risks of its use.
   1. Strongly Agree
   2. Somewhat Agree
   3. Neutral
   4. Somewhat Disagree
   5. Strongly Disagree
3. My graduate medical training (residency/fellowship) provided adequate training regarding the use of AI in healthcare, including the benefits and risks of its use.
   1. Strongly Agree
   2. Somewhat Agree
   3. Neutral
   4. Somewhat Disagree
   5. Strongly Disagree
4. I am concerned that the use of AI- and ML-tools will endanger patient safety.
   1. Strongly Agree
   2. Somewhat Agree
   3. Neutral
   4. Somewhat Disagree
   5. Strongly Disagree
5. I am concerned that AI tools will negatively impact the confidentiality of protected health information (PHI).
   1. Strongly Agree
   2. Somewhat Agree
   3. Neutral
   4. Somewhat Disagree
   5. Strongly Disagree
6. AI tools will improve personalized screening decisions for patients at risk for colorectal cancer.
   1. Strongly Agree
   2. Somewhat Agree
   3. Neutral
   4. Somewhat Disagree
   5. Strongly Disagree
7. AI tools will improve personalized screening decisions for patients at risk for breast cancer.
   1. Strongly Agree
   2. Somewhat Agree
   3. Neutral
   4. Somewhat Disagree
   5. Strongly Disagree
8. AI tools will improve personalized screening decisions for patients at risk for lung cancer.
   1. Strongly Agree
   2. Somewhat Agree
   3. Neutral
   4. Somewhat Disagree
   5. Strongly Disagree
9. I am concerned that AI tools will decrease the number of primary care attending physician, NP, or PA job positions available in the United States.
   1. Strongly Agree
   2. Somewhat Agree
   3. Neutral
   4. Somewhat Disagree
   5. Strongly Disagree
10. Who should bear responsibility for regulating the use and efficacy of AI- and ML-tools in cancer screening (check all that apply)?
    1. The U.S. federal government
    2. State governments
    3. Individual hospital systems
    4. Physician organizations
    5. Software developers
    6. Individual providers
    7. Other: ________
11. AI tools will __________ the number of *unnecessary* colonoscopies for patients at risk for colorectal cancer.
    1. significantly increase
    2. slightly increase
    3. have no significant effect on
    4. slightly decrease
    5. significantly decrease
12. AI- and ML-tools will __________ the number of *unnecessary* breast biopsies for patients at risk for breast cancer.
    1. significantly increase
    2. slightly increase
    3. have no significant effect on
    4. slightly decrease
    5. significantly decrease
13. AI- and ML-tools will __________ the number of *unnecessary* lung nodule biopsies for patients at risk for lung cancer.
    1. significantly increase
    2. slightly increase
    3. have no significant effect on
    4. slightly decrease
    5. significantly decrease
14. AI- and ML-tools will __________ the overall costs of colorectal cancer screening in the United States.
    1. significantly increase
    2. slightly increase
    3. have no significant effect on
    4. slightly decrease
    5. significantly decrease
15. AI- and ML-tools will __________ the overall costs of breast cancer screening in the United States.
    1. significantly increase
    2. slightly increase
    3. have no significant effect on
    4. slightly decrease
    5. significantly decrease
16. AI- and ML-tools will __________ the overall costs of lung cancer screening in the United States.
    1. significantly increase
    2. slightly increase
    3. have no significant effect on
    4. slightly decrease
    5. significantly decrease
17. I am concerned that AI- and ML-tools will decrease the number of primary care attending physician job positions available in the United States.
    1. Strongly Agree
    2. Agree
    3. Neutral
    4. Disagree
    5. Strongly Disagree

______________________________________________________________________________

**Section 3: Clinical Vignettes**

1. **Which of the options on this page would you choose?**

*You are a primary care provider who has just learned that your health system is deciding between purchasing a license to one of three FDA-approved AI-based tools for****colorectal cancer****screening management. This tool only applies to patients who have received at least one colonoscopy in the past. Assume that all tools are trained on the same initial dataset and have comparable accuracy.*

1. 5-year probability of colorectal cancer diagnosis ([0-100%] with 95% CI)
2. Recommended time until next colonoscopy (years, with 95% CI)
3. Binary recommendation of 1) colonoscopy **now** or 2) defer until next USPSTF-recommended screening
4. I would not use any of these tools
5. **Which of the options on this page would you choose?**

*You are a primary care provider who has just learned that your health system is deciding between purchasing a license to one of three FDA-approved AI-based tools for****breast cancer****screening management. This tool only applies to patients who have received at least one screening mammogram in the past. Assume that all tools are trained on the same initial dataset and have comparable accuracy.*

1. 5-year probability of breast cancer diagnosis ([0-100%] with 95% CI)
2. Recommended time until next mammogram (years, with 95% CI)
3. Binary recommendation of 1) mammogram **now** or 2) defer until next USPSTF-recommended screening
4. Segmentation of any suspicious masses on previous mammograms
5. I would not use any of these tools
6. **Which of the options on this page would you choose?**

*You are a primary care provider who has just learned that your health system is deciding between purchasing a license to one of three FDA-approved AI-based tools for****lung cancer****screening management. This tool only applies to patients who have received at least one low-dose chest CT in the past. Assume that all tools are trained on the same initial dataset and have comparable accuracy.*

1. 5-year probability of lung cancer diagnosis ([0-100%] with 95% CI)
2. Recommended time until next low-dose chest CT (years, with 95% CI)
3. Binary recommendation of 1) low-dose chest CT **now** or 2) defer until next USPSTF-recommended screening
4. Segmentation of any suspicious masses on previous low-dose chest CT
5. I would not use any of these tools

For the following questions, a "**care manager"** refers to a non-physician licensed healthcare provider who can directly coordinate additional lab work or imaging studies with a patient.

1. **Which of the options on this page would you choose?**

*You are a primary care provider who has just learned that your health system is purchasing a license for a new tool that estimates the five-year risk of****colorectal cancer****([0-100%] with 95% CI) for each patient above the age of 40, regardless of previous colonoscopy history. The health system is deciding how best to implement the tool in clinical workflow. Please select how you would prefer to implement the tool's predictions.*

1. Automatically generate an Electronic Health Record (EHR) chart flag for each eligible patient
2. Have an easily accessible risk calculator hosted online or through a smartphone application
3. Provide a list of predictions to the practice’s licensed care manager once per year
4. None of the above
5. **Which of the options on this page would you choose?**

*You are a primary care provider who has just learned that your health system is purchasing a license for a new tool that estimates the five-year risk of****breast cancer****([0-100%] with 95% CI) for each patient above the age of 40, regardless of previous mammogram history. The health system is deciding how best to implement the tool in clinical workflow. Please select how you would prefer to implement the tool's predictions.*

1. Automatically generate an Electronic Health Record (EHR) chart flag for each eligible patient
2. Have an easily accessible risk calculator hosted online or through a smartphone application
3. Provide a list of predictions to the practice’s licensed care manager once per year
4. None of the above
5. **Which of the options on this page would you choose?**

*You are a primary care provider who has just learned that your health system is purchasing a license for a new tool that estimates the five-year risk of****lung cancer****([0-100%] with 95% CI) for each patient above the age of 50 with a smoking history. The health system is deciding how best to implement the tool in clinical workflow. Please select how you would prefer to implement the tool's predictions.*

1. Automatically generate an Electronic Health Record (EHR) chart flag for each eligible patient
2. Have an easily accessible risk calculator hosted online or through a smartphone application
3. Provide a list of predictions to the practice’s licensed care manager once per year
4. None of the above
5. Your hospital system has recently purchased a license to an AI-based tool that segments (outlines) regions of low-dose chest CTs that are **suspicious for pathologic lung nodules**. The tool offers three options for how suspicious nodules can be communicated to the healthcare team. Assume the patient has never been diagnosed with a cancer. Please select which members of the care team should be alerted to suspicious nodules (select all that apply).
6. Alert the attending radiologist who read the CT scan
7. Alert the patient’s PCP
8. Alert the case manager assigned to the PCP’s practice
9. None of the above
10. Your hospital system has recently purchased a license to an AI-based tool that segments (outlines) regions of screening mammograms that are **suspicious for pathologic breast masses**. The tool offers three options for how suspicious masses can be communicated to the healthcare team. Please select the option you would prefer in terms of the communication of the tool. Please select which members of the care team should be alerted to suspicious nodules (select all that apply).
11. Alert the attending radiologist who read the CT scan
12. Alert the patient’s PCP
13. Alert the case manager assigned to the PCP’s practice
14. None of the above
15. You are a primary care provider who is deciding between the following options for an AI-based tool to aid in the screening of at-risk patients for **lung cancer**. The tool will receive a low-dose chest CT with lung cancer screening protocol for at-risk patients and report whether it detects a nodule that should receive follow-up (either through increased surveillance, biopsy, or surgical consultation). Each option has a different method of implementation in the clinical workflow.
16. Attending radiologist may view AI-generated report for a given study before signing her final report. Both reports are available in the EMR.
17. Attending radiologist signs her final radiology report before AI-generated report can be viewed. Both reports are available in the EMR.
18. Attending radiologist views AI-generated report for a given study before viewing study images herself. Attending then views images and signs the final report. Both reports are available in the EMR.
19. None of the above
20. You are a primary care provider who is deciding between the following options for an AI-based tool to aid in the screening of at-risk patients for **breast cancer**. The tool will receive a screening mammogram for at-risk patients and report whether it detects a nodule that should receive follow-up (either through increased surveillance, biopsy, or surgical consultation). Each option has a different method of implementation in the clinical workflow.
21. Attending radiologist may view AI-generated report for a given study before signing her final report. Both reports are available in the EMR.
22. Attending radiologist signs her final radiology report before AI-generated report can be viewed. Both reports are available in the EMR.
23. Attending radiologist views AI-generated report for a given study before viewing study images herself. Attending then views images and signs the final report. Both reports are available in the EMR.
24. None of the above
25. The “explainability” of AI models is an important factor in determining provider trust of these models’ recommendations. You are a primary care provider who is piloting a new tool to aid in the management of **breast cancer** screening for patients who recently received a standard mammogram. The tool provides a binary recommendation (follow-up needed or no follow-up needed). The developers of this tool want to know the best way in which the tool can explain its decision-making. They provide you three options for how to accomplish this. Visual accompaniments are below.
26. Reports the percent probability (0-100% with 95% CI) of developing breast cancer in the next five years. This is accompanied by a bar graph displaying the risk factors that contribute most to an individual patient’s risk for breast cancer
27. Estimates volumetric breast density from mammogram ([0-100%] with 95% CI) by segmenting fatty, semi-fatty, and dense breast regions. From this, a BI-RADS density category is assigned.
28. Segments suspicious masses in mammograms.
29. I have no preference for any of these explainability methods.

Option A:

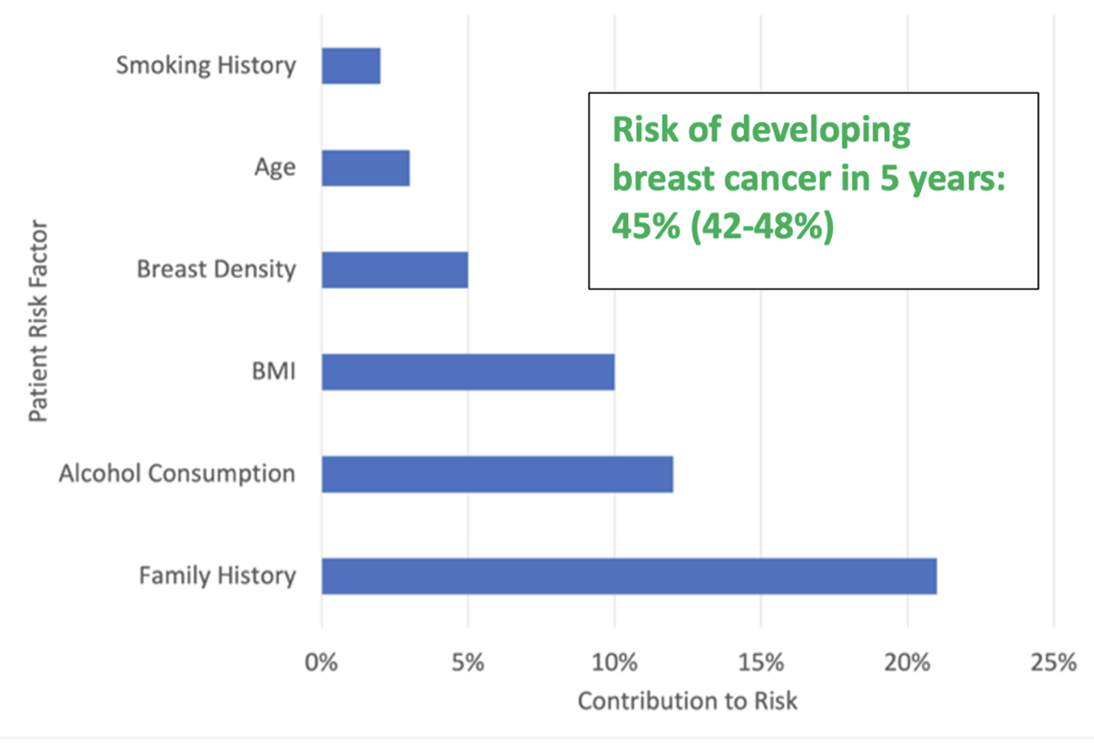


Option B:
Mammographic segmentation of fatty (navy), semi-fatty (grey), semi-dense (yellow), and dense (red) breast tissue (figure is reproduced with permission from He et al.)**.**

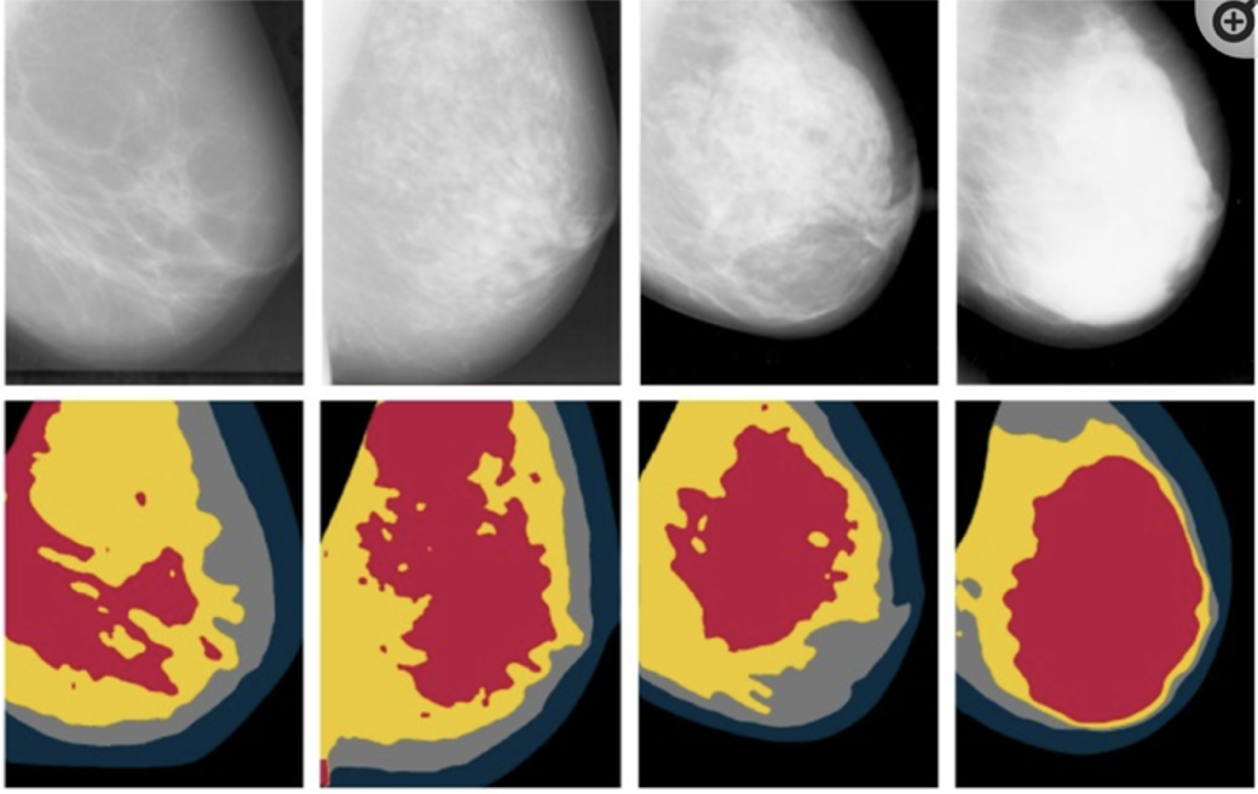


Option C:
Mammographic segmentation of masses (blue line) suspicious for breast cancer (figure is reproduced with permission from Hassan et al. © 2014 IEEE).


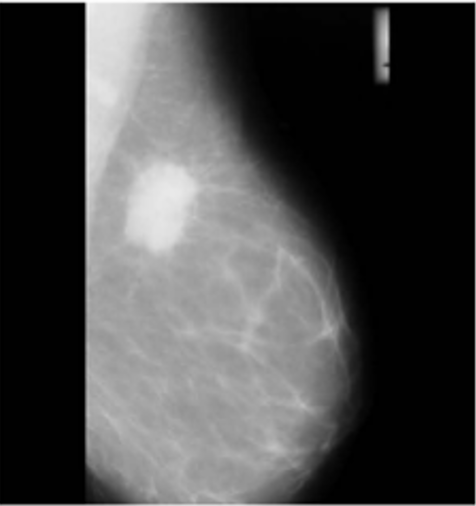

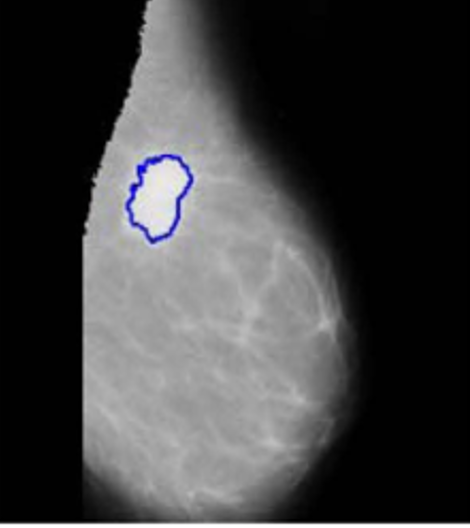

Supplement: sj-docx-1-mpp-10.1177_23814683251329007 – Supplemental material for Primary Care Provider Preferences Regarding Artificial Intelligence in Point-of-Care Cancer Screening [file sj-docx-1-mpp-10.1177_23814683251329007.docx]
